# A predictive model for the transformation from cervical inflammation to cancer based on tumor immune-related factors

**DOI:** 10.3389/fimmu.2025.1532048

**Published:** 2025-04-25

**Authors:** Wenjie Wang, Chuntao Huang, Shiwen Bi, Huiting Liang, Songlin Li, Tingting Lu, Ben Liu, Yong Tang, Qi Wang

**Affiliations:** ^1^ Department of Experimental Research, Guangxi Medical University Cancer Hospital, Nanning, Guangxi, China; ^2^ Key Laboratory of Early Prevention and Treatment of Regional High-incidence Tumors, Ministry of Education Key Laboratory, Guangxi Medical University, Nanning, Guangxi, China; ^3^ University Engineering Research Center of Oncolytic & Nanosystem Development, Nanning, Guangxi, China; ^4^ Institute of Life Sciences, Guangxi Medical University, Nanning, Guangxi, China; ^5^ Department of Pathology, Wuming Hospital of Guangxi Medical University, Nanning, Guangxi, China; ^6^ State Key Laboratory of Targeting Oncology, Guangxi Medical University, Nanning, Guangxi, China

**Keywords:** cervical intraepithelial neoplasia (CIN), cervical cancer (CC), tumor immune microenvironment (TIME), tumor-infiltrating immune cells (TICs), tumor-infiltrating cell-related genes (TICRGs), multiplexed immunohistochemistry (mIHC), random forest, predictive model

## Abstract

**Introduction:**

Persistent high-risk human papillomavirus (HR-HPV) infection is crucial in transforming cervical intraepithelial neoplasia (CIN) into cervical cancer (CC) by evading immune responses. Additionally, changes in the tumor immune microenvironment (TIME) are increasingly linked to CIN progression to CC.

**Methods:**

In this study, we used public databases to collect transcriptome data for CIN, CC, and normal cervix, employing LASSO regression to find TIP genes with differential expression. We also used the CIBERSORT algorithm to analyze immune cells in the cervix. ROC curves were plotted to assess tumor-infiltrating immune cells (TICs) and the expression of tumor-infiltrating cell-related genes (TICRGs) for predicting CC efficacy and identifying immune-related genes and cells associated with cervical disease progression for future modeling. We developed a cervical "inflammation-cancer transition" prediction model using the random forest algorithm and assessed its accuracy with internal and external data. Clinical samples from two hospitals were analyzed using multiplexed immunohistochemistry (mIHC) to detect risk factors in various cervical diseases, serving as an independent validation cohort for the model's reliability.

**Results:**

Four genes, ARG2, HSP90AA1, EZH2, ICAM1, and two immune cells, M1 macrophages and activated CD4 memory T cells, were selected as variables, and a predictive model was constructed. The model achieved an AUC of 1 for internal training sets and 0.912 for testing sets. For validation cohort, the AUC was 0.864 for GSE7803 and 0.918 for TCGA/GTEx. For external validation (GSE39001, GSE149763, and GSE138080), the AUC was 0.703, 0.889 and 0.696. At the same time, the mIHC experimental results also effectively validated the stability of the model.

**Discussion:**

In conclusion, the developed model enhances the predictive accuracy for the progression of CIN to CC and offers novel insights for the early diagnosis and screening of CC.

## 1 Introduction

Cervical cancer (CC) ranks as the fourth leading cause of cancer-related mortality among women, with approximately 604,127 new cases and 341,831 deaths reported globally in 2020 (1). Despite the consistently high incidence of CC, it remains a preventable disease. Early diagnosis and timely intervention can effectively prevent tumor development and progression.

It is well-established that persistent infection with high-risk human papillomavirus (HR-HPV) represents a significant contributing factor in the progression of cervical intraepithelial neoplasia (CIN) to CC (2, 3). Nevertheless, it is estimated that approximately 85% to 90% of women infected with HPV achieve spontaneous viral clearance through the body’s immune response, while only 10% to 15% experience persistent infection (4, 5). Consequently, it can be hypothesized that the progression to CC may depend on the presence of additional cofactors (6). Previous studies have shown that demonstrated that cells infected with the HPV play a crucial role in creating a supportive and immunosuppressive post-infection microenvironment (PIM), which promotes viral persistence and replication by interacting with normal resident cells (7, 8). The chronic inflammatory response elicited by persistent HPV infection leads to recurrent local tissue injury and regeneration in the cervix. The accumulation of various cellular damage events ultimately contributes to the progression from CIN to CC (9, 10). Conversely, persistent HPV infection is significantly linked to modifications in the tumor immune microenvironment (TIME) (11). Several studies have suggested that an imbalance of local immune cells within the cervix may facilitate persistent HPV infection (12), with particular emphasis on the dysregulation of CD4+ and CD8+ T cell populations. The CD4+ T cell subset plays a pivotal role in anti-tumor immunity, tumor immune evasion, tolerance mechanisms, TIME and the maintenance of immune homeostasis (13). During the primary immune period following HPV infection, CD4+T cells are activated in secondary lymphoid organs, enhancing cellular or humoral immune responses to eliminate pathogens through the action of T-helper 1 (Th1) and T-helper 2 (Th2), respectively (14, 15). Dysfunctional CD4+T cells have a weaker ability to clear viruses, while the recruitment and expansion of regulatory T cells (Tregs) create a favorable immunosuppressive environment for HPV (16), leading to the long-term presence of HPV and increasing the risk of cervical disease progression and malignant transformation.

Studies indicate that the immune system has a dual role in cancer: it can both eliminate cancer cells and promote tumor growth by creating a supportive microenvironment (17, 18). As CC advances, it has the potential to create an immunosuppressive microenvironment, thereby undermining the host’s anticancer immune response. The phenomenon of immune escape is intricately linked to alterations in tumor-infiltrating immune cells (TICs) and the expression of tumor-infiltrating cell-related genes (TICRGs) within the tumor microenvironment of CC. For instance, prior research has demonstrated that the progression of CC is frequently associated with an elevated presence of regulatory T cells (Tregs) and an upregulation of the CTLA-4 gene expression (19, 20). Therefore, exploring key immune factors in cervical inflammation-cancer transformation is crucial for developing a CC predictive model. Recently, more molecules important for CC development and prognosis have been identified (21–23).

This study employed the Random Forest algorithm on public transcriptomic data to identify crucial immune factors in the cervical “inflammation-cancer transition” and create a predictive model, validated with internal and external data. multiplexed immunohistochemistry (mIHC) (24) was used to assess immune-related gene and cell expression in clinical samples, confirming the model’s reliability. Our goal is to analyze the gene expression levels and immune cell infiltration status in HPV-infected patients. This analysis will help assess their likelihood of developing cervical cancer, enabling early diagnosis and treatment by clinicians.

## 2 Materials and methods

### 2.1 Data collection

#### 2.1.1 Public database data collection

The datasets GES63514, GSE7803, GSE39001, GSE149763 and GSE138080 were obtained from the Gene Expression Omnibus (GEO) repository. Specifically, the GSE63514 dataset includes 24 normal cervical samples, 62 CIN samples, and 28 CC samples, the GSE7803 dataset contains 10 cases of normal cervical samples, 7 CIN samples, and 21 CC samples, while the external verification queue (GSE39001, GSE149763 and GSE138080) contains 22 normal cervical samples, 18 CIN samples, and 56 CC samples. Additionally, the transcriptomic data and clinical information of 306 cases of CC and 13 cases of normal cervical tissues were acquired from The Cancer Genome Atlas (TCGA) and the Genotype-Tissue Expression (GTEx) databases. The GSE63514 dataset was used to train the “inflammation-cancer transformation” model, and its accuracy was validated using the GSE7803 and TCGA/GTEx datasets, as detailed in **Supplementary Table 1**.

#### 1.1.2 Collection clinical data and tissue samples

This study used 31 paraffin-embedded CC samples from untreated patients at Guangxi Medical University Cancer Hospital and who had not undergone any other treatments. These patients had their first cervical resection with a pathologically confirmed CC diagnosis between 2016 and 2018. The samples used were approved by the Ethics Committee of Guangxi Medical University Cancer Hospital (No. KY2024560). Pathological sections comprising 22 CIN samples and 21 normal cervical samples were procured from Wuming Hospital of Guangxi Medical University, with ethical clearance granted by the Ethics Committee of Wuming Hospital of Guangxi Medical University (Approval No. WM-2024(218)). (See **Supplementary Table 2**).

### 2.2 Screening of TICRG predictors and TIC predictors

#### 2.2.1 LASSO regression screening of TICRGs

From the Tumor Immunophenotyping (TIP) database, 178 TICRGs were analyzed. Using the GSE63514 cohort, 166 of these genes were identified as potential candidates, with 28 CC patients as the positive group and 62 CIN patients as the negative group (**Supplementary Figure 1A**). The 166 TICRGs underwent down-conversion using Least Absolute Shrinkage and Selection Operator (LASSO) regression (25, 26) via the “glmnet” R package. The model was cross-validated and run 1,000 times, with λ= 0.01258, achieving the highest model Area Under the Curve (AUC) value. This process identified 31 TICRGs with non-zero and correlated coefficients could be identified, as illustrated in **Supplementary Figure 1** and **Table 3**.

GraphPad Prism (version 8.0.2) was used to create receiver operating characteristic (ROC) curves for the 31 TICRGs with non-zero coefficients in the GES63514, GSE7803, and TGGA datasets. CC samples served as positive controls, while normal cervix/CIN II/CIN III samples were negative controls. TICRGs showing significant progression from CIN to CC were identified with criteria of P < 0.05 and AUC > 0.6. Statistically significant TICRGs that are common across the three datasets will be incorporated into the model. We define the filtered TICRGs as TICRG predictors.

Using LM22 as a reference matrix, the CIBERSORT (27) algorithm analyzed raw gene expression data from the GSE63514, GSE7803, TCGA/GTEx, GSE39001, GSE149763, and GSE138080 cohorts to identify 22 TICS profiles. ROC curves were generated with GraphPad Prism (8.0.2) to compare these profiles across datasets. Each cohort was assessed for significant TICS progression from CIN to CC, using criteria of P < 0.05 and AUC > 0.6, with CC samples as positive controls and normal cervix/CIN samples as negative controls. The datasets GSE63514, GSE7803, and TCGA/GTEx included significant TICS predicting “inflammation-cancer transformation”, named TIC predictors, used for further modeling.

### 2.3 Development of the predictive model for cervical “inflammation-cancer transformation”

The predictive model used 28 CC samples as positive controls and 76 CIN/normal samples as negative controls from the GSE63514 cohort. Expression profiles of specific genes and cell types were divided into training and validation sets at various ratios. A Random Forest algorithm was employed, with the 7:3 ratio model proving to be the most optimal. The program was configured with 500 trees, and stability was achieved with more than 250 trees. The ROC curve was plotted, and the model’s AUC was calculated for evaluation. Validation cohort was performed using the GSE7803 and TCGA/GTEx cohorts, along with experimental data. Variables were analyzed for expression differences across disease stages, and Pearson’s correlation analysis was conducted on the model’s variables (28). Logistic regression and Pearson’s correlation analyses were also performed on the data from the experimental cohort.

### 2.4 mIHC assay

Six variables were analyzed in 74 clinical samples using mIHC with TSA. Cervical tissues, embedded in paraffin and sectioned at 5 μm, were deparaffinized, dehydrated, and underwent high-pressure antigen retrieval with 1 mM Tris-EDTA buffer (pH 9.0) for 18 min. The samples were blocked using blocking solution (Beyotime, Cat. P0102). The primary and secondary antibodies were applied, incubated at 37°C for 2 hours, and rinsed three times with PBS, followed by TBST washing. A 1:100 diluted PPD520 TSA fluorescent dye (PANOVUE, Cat.10005100100) in TSA signal amplification solution (PANOVUE, Cat.10021001050), was added. The secondary antibody and second fluorescent stain (PPD570 or PPD650, PANOVUE, Cat.10008100100 or Cat.10010100100) were added following the same procedure as the first antibody. Detection of ICAM1 monoclonal antibody (ZenBio, Cat.R24650), HSP90AA1 monoclonal antibody (ZenBio, Cat.R24635), ARG2 polyclonal antibody (ZenBio, Cat.R389341), EZH2 monoclonal antibody (ZenBio, Cat.R24813), CD68 polyclonal antibody (ZenBio, Cat.250019), CD163 monoclonal antibody (ZenBio, Cat.R50062), iNOS polyclonal antibody (ZenBio, Cat.340668), CD4 monoclonal antibody (ZenBio, Cat.R50028), CD44 monoclonal antibody (ZenBio, Cat.R50120), CD206 monoclonal antibody (ZenBio, Cat.R51183), and CD45RO (Santa Cruz Biotechnology, Cat.sc-1183) expression. After fluorescence staining, nuclei were stained with a 1:500 DAPI solution (Solarbio, Cat.C0060) in PBS for 10 min at room temperature, and slices were sealed with enhanced antifluorescence quenching sealer (PANOVUE, Cat. 10022001010).

Tissue samples were imaged with a microimaging system (Tissue Gnostics, Austria) using a 20× objective lens across four channels: DAPI, FITC, Texas Red, and CY5. Fluorescence was quantitatively analyzed with the Strata Quest application. Sections were analysis by selecting 3–8 random regions of interest (ROIs) sized 0.75×0.75 from each section. After adjusting the ROI parameters, the density of positive proteins (No./mm²) in each ROI was calculated after the completion of the quantitative analysis.

### 2.5 Statistical analysis

Statistical analysis was performed using GraphPad Prism (8.0.2) and R Studio (4.4.1) appropriate software packages. The Wilcoxon rank-sum test compared two groups, while the Kruskal-Wallis test was used for multiple samples. Group comparisons for measures employed the chi-square test, with a P value of <0.05 indicating statistical significance.

## 3 Results

### 3.1 Screening TICRG predictors for CC occurrence

**Figure 1:**
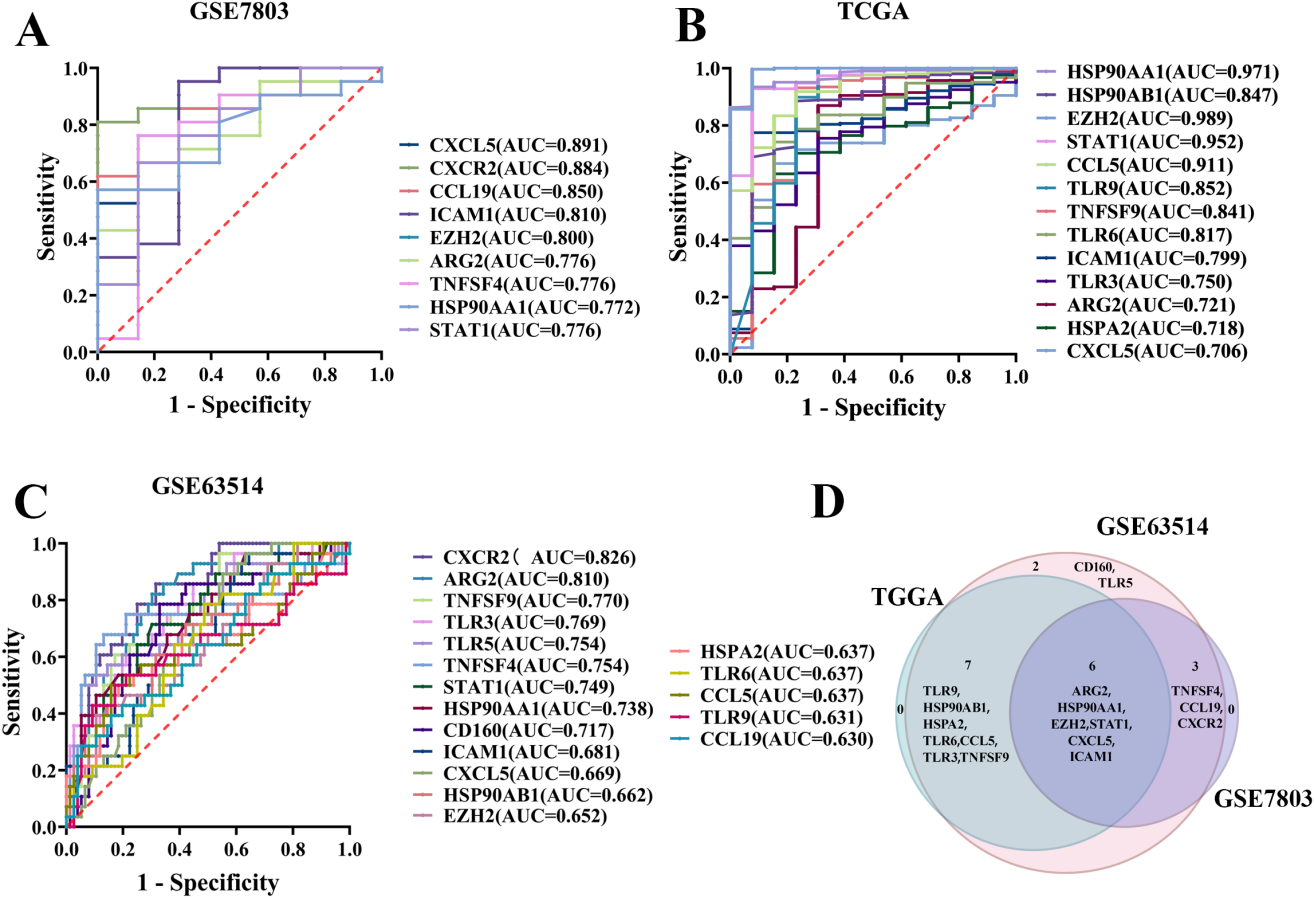
Screening of TICRG predictors for the occurrence of CC. **(A)** The ROC curve analysis demonstrated statistical significance for the prediction of CC using single TICRG in the GSE7803 cohort. **(B)** Statistically significant ROC curves were observed for single TICRG predictions of CC in the TCGA/GTEx cohort. **(C)** The GSE63514 cohort exhibited statistically significant ROC curves for the prediction of CC using single TICRG. **(D)** The Venn diagram illustrates TICRG predictors that are shared among the three datasets.

ROC curves were generated for each of the 31 TICRGs with non-zero coefficients from the GSE7803, TCGA/GTEx, and GSE63514 cohorts. From these analyses, 18, 9, and 13 TICRG**s** were identified in the respective cohorts as statistically significant predictors of CC occurrence, based on the criteria of an Area Under the Curve (AUC) greater than 0.6 and a p-value less than 0.05 (**Figures 1A–C**). Notably, six TICRG predictors—ARG2, HSP90AA1, EZH2, STAT1, CXCL5, and ICAM1—were consistently identified across all three datasets as significant individual predictors of CC (**Figure 1D**).

### 3.2 Screening TIC predictors to predict cervical carcinogenesis

**Figure 2:**
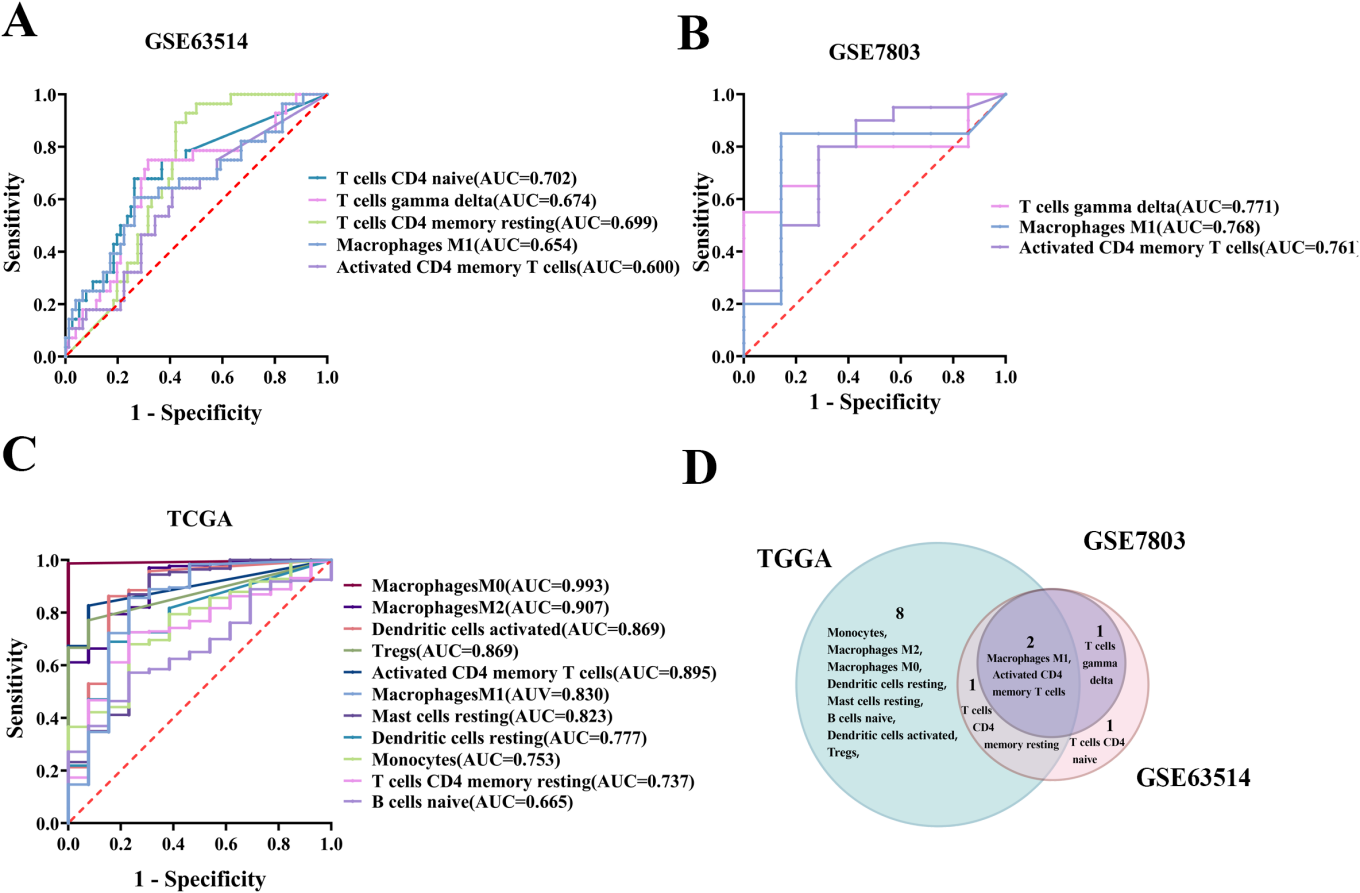
Screening of TIC predictors for their predictive potential in cervical carcinogenesis. **(A)** Significant ROC curves for individual TICs predicting CC in the GSE63514 cohort. **(B)** Significant ROC curve for single immune cell prediction in the GSE7803 cohort. **(C)** Significant ROC curve for individual TICs in the TCGA/GTEx cohort. **(D)** Venn diagram showing TIC predictors significantly predicting cervical carcinogenesis based on three datasets.

Gene expression data from the GSE63514, GSE7803, and TCGA/GTEx cohorts were analyzed utilizing the CIBERSORT algorithm to identify 22 distinct immune cell profiles. ROC curves were subsequently generated for these TICs, revealing that 4, 3, and 11 cell types from the respective cohorts significantly predicted CC occurrence, as indicated by an area under the curve (AUC) greater than 0.6 and a p-value less than 0.05 (**Figures 2A–C**). Additionally, across the three datasets GSE63514, GSE7803, and TCGA/GTEx, macrophage M1 and activated CD4 memory T cells were identified as individual predictors of CC with statistical significance (**Figure 2D**).

### 3.3 Expression and correlation analysis of five TICRG predictors and two TIC predictors

A comprehensive statistical analysis was performed on datasets from the GSE63514, GSE7803, and TCGA/GTEx cohorts, focusing on the expression levels of ARG2, HSP90AA1, EZH2, STAT1, ICAM1, macrophage M1, and activated CD4 memory T cells. The findings revealed a consistent increase in the expression of HSP90AA1, EZH2, STAT1, ICAM1, macrophage M1, and activated CD4 memory T cells in correlation with disease progression across all three cohorts. ARG2 shows a decreasing trend.

**Figure 3:**
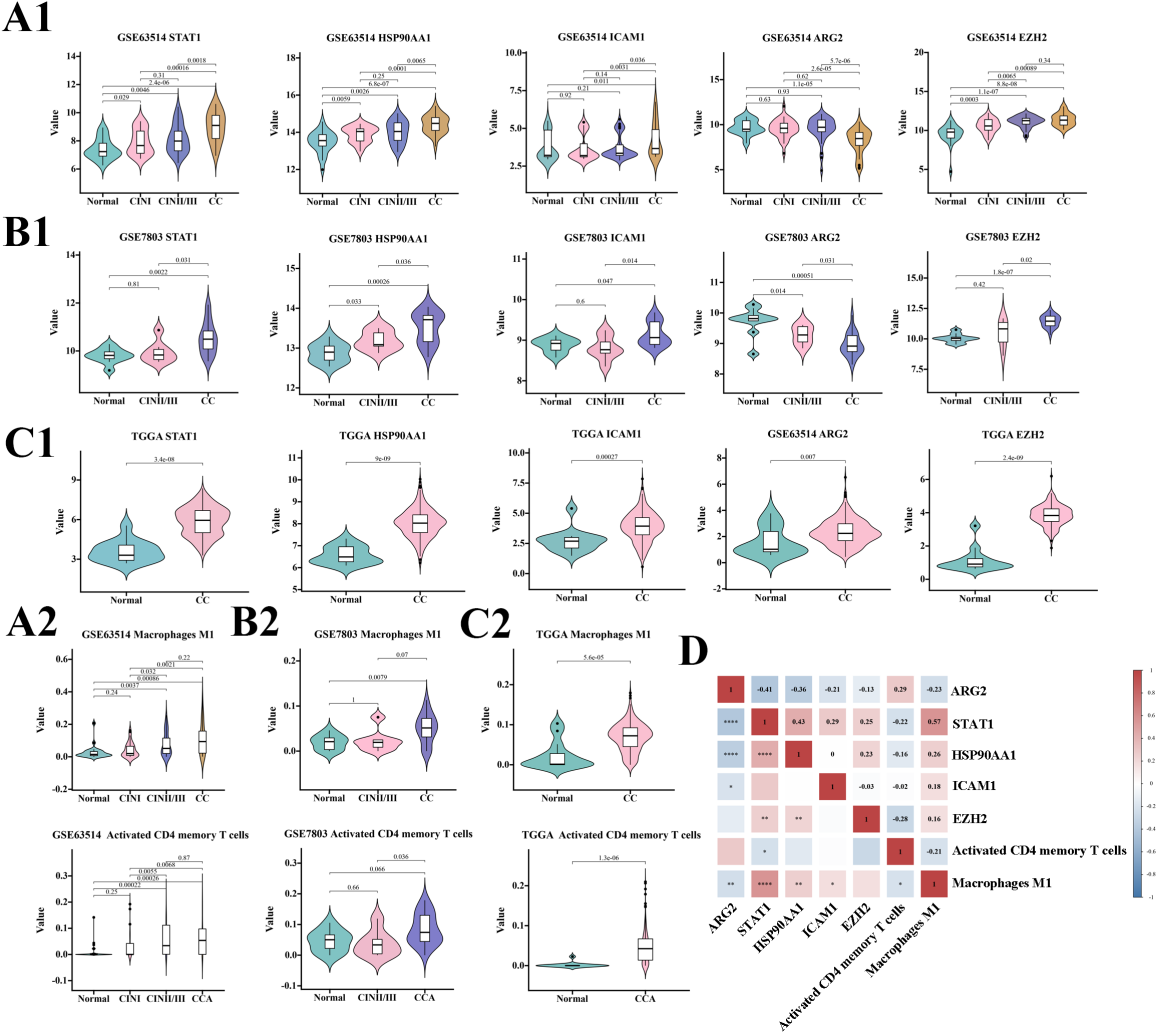
Predictive potential of each variable for cervical cancer across three cohorts and a correlation analysis. Panels **(A1, A2)** depict the expression of seven variables within the GSE63514 cohort at various stages of cervical disease. Similarly, panels **(B1, B2)** present the expression of these variables in the GSE7803 cohort, while panels **(C1, C2)** display the expression in the TGGA cohort, each at different stages of cervical disease. Panel **(D)** provides a correlation analysis of the seven variables within the predictive model, with significance levels indicated as follows: *P<0.05, **P<0.01, ***P<0.001, ****P<0.0001.

Among the five TICRG predictors, HSP90AA1 showed the highest expression across all lesion stages, followed by EZH2, while ICAM1 had the lowest expression (**Figure 3**). Correlation analysis in the GSE63514 cohort revealed a negative correlation between ARG2 and STAT1, HSP90AA1, ICAM1, and macrophage M1, but a positive correlation with activated CD4 memory T cells. The strongest correlation was between ARG2 and STAT1 (-0.41, P < 0.0001), followed by HSP90AA1 (-0.36, P < 0.0001). STAT1 positively correlated with HSP90AA1, macrophage M1, ICAM1, and EZH2, and negatively with ARG2 and activated CD4 memory T cells. Its strongest link was with macrophage M1 (0.57, P < 0.0001), leading to STAT1’s exclusion from the model. Activated CD4 memory T cells negatively correlated with EZH2, STAT1, ICAM1, and macrophage M1 (**Figure 3D**).

### 3.4 Development of a predictive model for the transformation from cervical inflammation to cancer

#### 3.4.1 Random forest algorithm for predicting CC transformation

**Figure 4:**
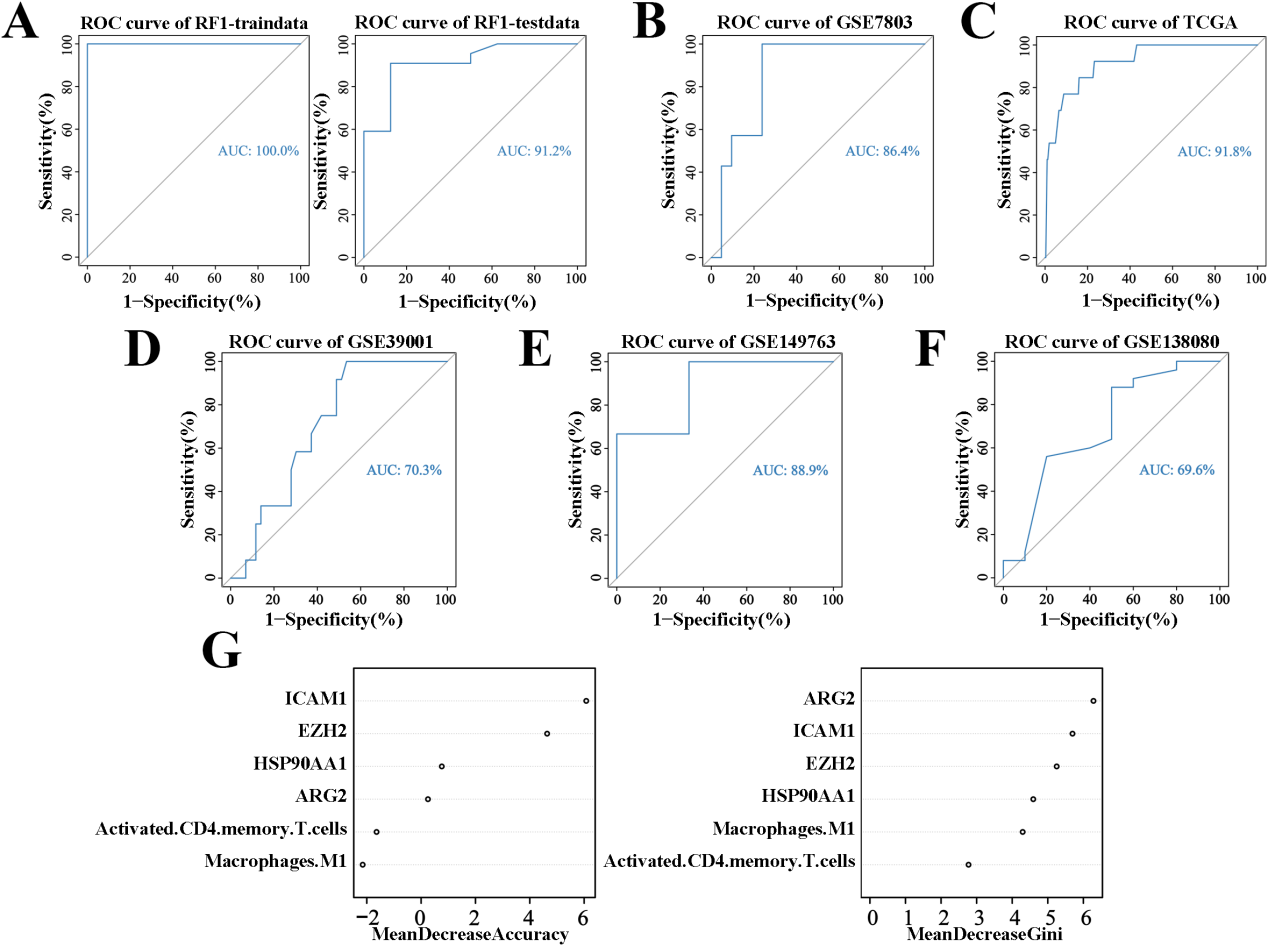
ROC curves and weights for predicting CIN to CC conversion using the random forest algorithm. **(A)** ROC curves for the cervical “inflammatory cancer transformation” model in training and test sets. **(B)** ROC curves for the same model in the GSE7803 cohort. **(C)** ROC curves for the TCGA/GTEx cohort. **(D)** ROC curves for the same model in the GSE39001 cohort. **(E)** ROC curves for the GSE149763 cohort. **(F)** ROC curves for the GSE138080 cohort. **(G)** Importance weights of six variables in the GSE63514 cohort’s prediction model.

The random forest model, built using the GSE63514 cohort, achieved an AUC of 1 in the training set and 0.912 in the test set (**Figure 4A**). The cervical “inflammation-cancer transition” prediction model’s accuracy was confirmed using the GSE7803 and TCGA/GTEx cohorts, showing AUCs of 0.864 and 0.918, respectively (**Figures 4B, C**). The accuracy of the cervical “inflammation cancer transition” prediction model was further confirmed using external cohorts GSE39001, GSE149763, and GSE138080, with AUCs of 0.703, 0.889 and 0.696 (**Figures 4D–F**). This suggests the random forest model effectively predicts CIN progression to CC. Six variables were included in the model, ranked by Gini coefficient. This analysis demonstrated that the ARG2 gene exhibited the highest weight ratio within the random forest model (**Figure 4G**).

### 3.5 mIHC assay confirms predictive model for cervical inflammation progression to cancer

#### 3.5.1 Six variables in a predictive model for cervical inflammation-cancer transition across various pathology stages

**Figure 6:**
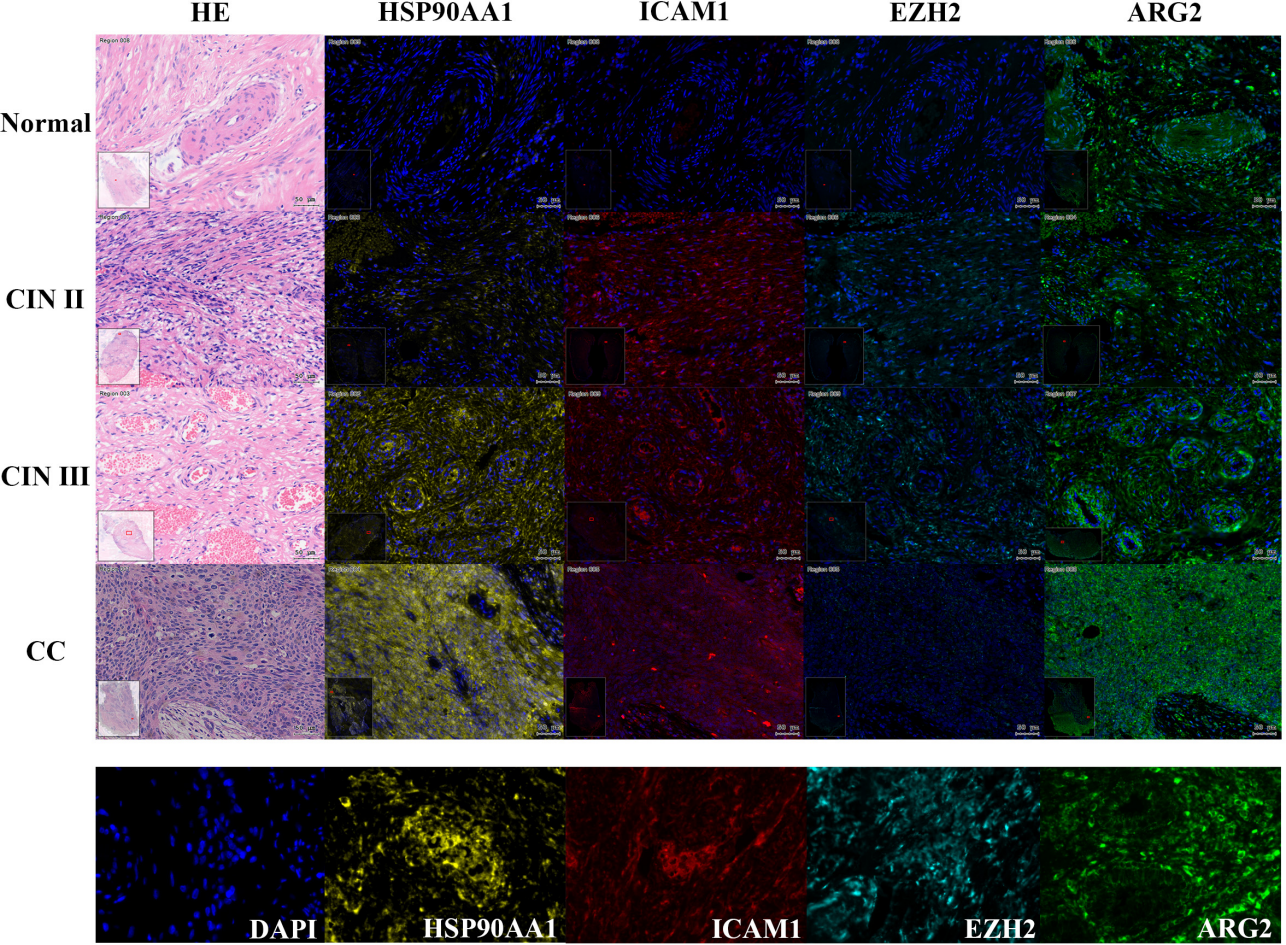
Expression of four TICRG predictors in mIHC experiments across various cervical disease stages. As shown in the example diagram, dark blue represents DAPI (nucleus), yellow represents HSP90AA1 protein expression, red represents ICAM1 protein expression, lake blue represents EZH2 protein expression, and green represents ARG2 protein expression. Calculate the expression level of each protein based on the ratio of its positive fluorescence value to DAPI. HSP90AA1, ICAM1, and ARG2 showed a significant upward trend in CIN III and CC stages (P=2.3e-3, P=0.09, P=0.05), while EZH2 showed a downward trend in CIN III and CC stages (P=4.9e-3).

**Figure 7:**
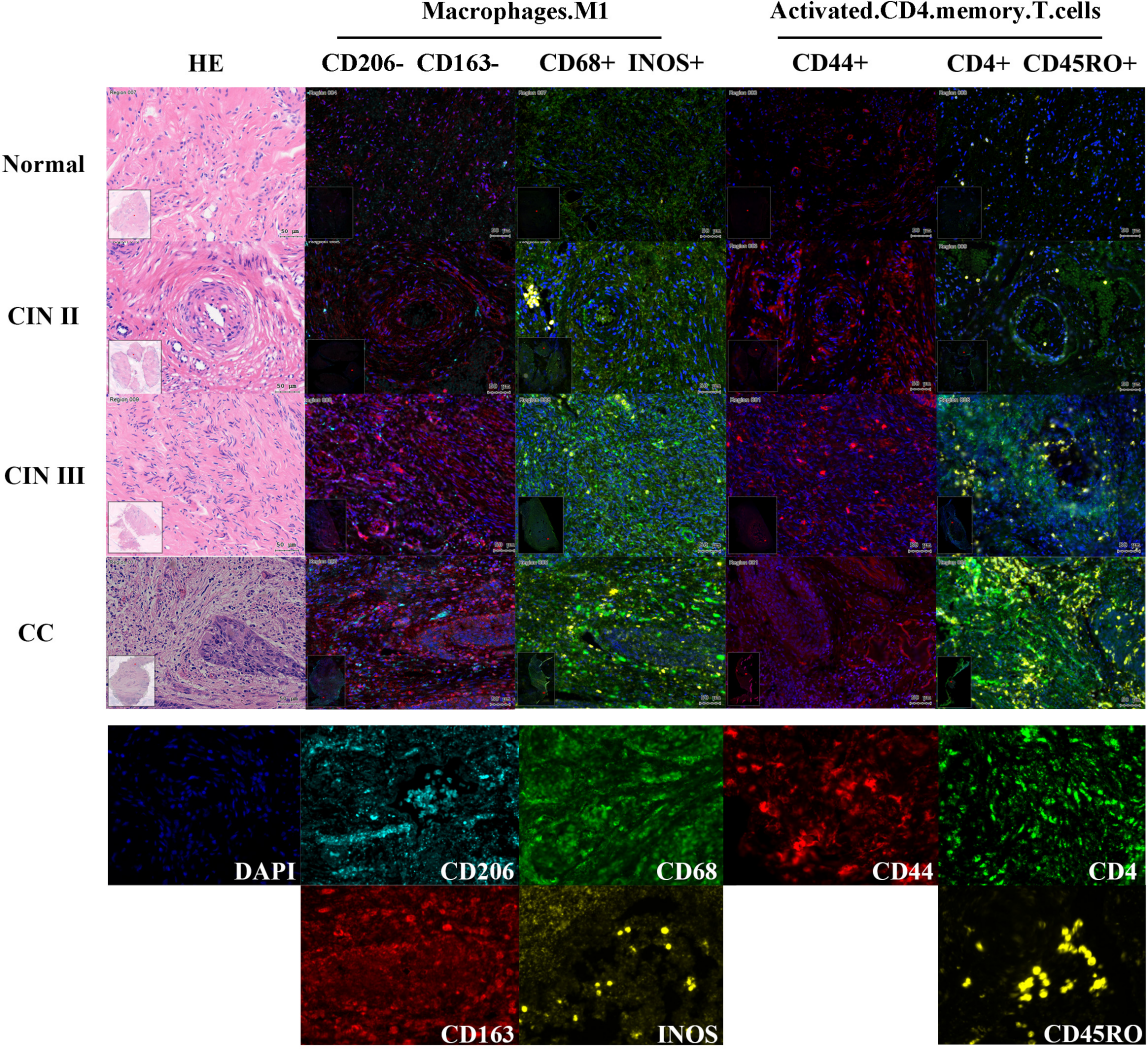
Expression of two TIC predictors in mIHC experiments across cervical disease stages. Define Macrophage M1 using CD206-, CD163-, CD68+, INOS+; Use CD206+, CD163+, CD68+to define Macrophage M2; Use CD4+, CD4+, CD45RO+to define activated CD4 memory T cells. As shown in the example diagram in the figure, dark blue represents DAPI (cell nucleus), and the expression level of each protein is calculated based on the ratio of its positive fluorescence value to DAPI. Macrophage M1 showed a significant upward trend in CIN III and CC stages (P=1.7e-4), while activated CD4 memory T cells showed an upward trend in CIN III and CC stages, but the trend was not significant (P=0.95).

To validate the predictive model for cervical “inflammation-cancer transformation,” we conducted mIHC analysis on six variables across 74 clinical paraffin-embedded samples. **Figure 6** presents the expression profiles of HSP90AA1, ICAM1, EZH2, and ARG2 in various stages of cervical pathology, including normal cervix, CIN II, CIN III, and CC. Macrophage M1 phenotype was characterized by the markers CD68+, INOS+, CD206-, and CD163-, whereas activated CD4 memory T cells were identified by the markers CD4+, CD44+, and CD45RO+. The distribution and expression of cell types across different stages of cervical disease are shown in **Figure 7**.

#### 3.5.2 The predictive model for the “inflammation-cancer transition” developed utilizing clinical cohort data and mIHC analyses, effectively anticipates the progression from CIN III to CC

**Figure 5:**
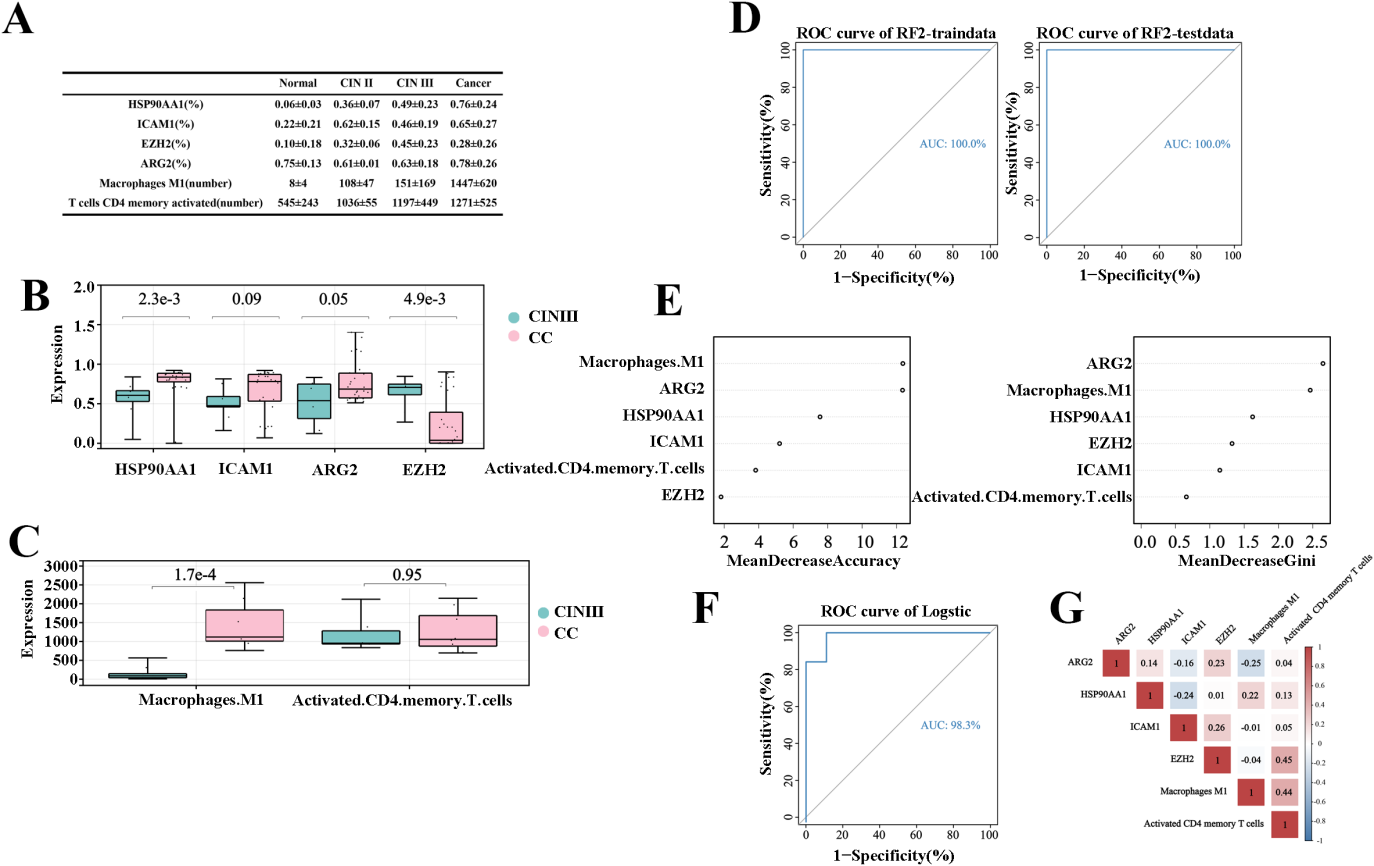
Prediction model for “inflammation-cancer transition” using clinical cohorts and mIHC data. **(A)** Expression levels of each variable in the “inflammation-cancer transformation” prediction model across cervical disease stages (% of individuals). **(B)** Four TICRG predictors protein levels in mIHC data at various cervical disease stages. **(C)** Activated CD4 memory T cells and M1 macrophage counts across different cervical disease stages. **(D)** ROC curves for predicting CIN progression to CC using a model based on cervical “inflammation-cancer transformation” data in training and test sets. **(E)** Importance of six variables in the “inflammation-cancer transformation” prediction model. **(F)** Predicting ROC curves for progression of CIN to CC using Logistic regression. **(G)** provides a correlation analysis of the six variables within the Logistic regression.

The experimental results were analyzed by comparing the percentage of cells expressing target genes to the total cell count or the number of gene-expressing cells in the sample tissues (**Figure 5A**). The study found that as cervical disease advanced, the protein expression of genes HSP90AA1, ICAM1, and ARGE increased, except for EZH2, as shown in **Figure 5B**. Similarly, Activated CD4 memory T cells and Macrophage M1 populations also rose with disease progression, as depicted in **Figure 5C**. Comparing CIN III and CC groups, significant differences were noted in HSP90AA1, ICAM1, EZH2, ARG2, and Macrophages M1 (P<0.01).These findings indicate a potential link between these variables and the progression from CIN III to CC.

The experimental results from six variables via mIHC served as an independent validation cohort. Using the method from section 2.3.1, a prediction model for “inflammation-cancer transition” was developed for 17 CIN III and 31 CC samples. ROC curves assessed the models, with the random forest model achieving an AUC of 1 in both the training and testing sets (**Figure 5D**). The prediction model for the “inflammation-cancer transition,” developed using the experimental cohort, demonstrated the highest weight ratio for ARG2, aligning with the findings from the training cohort (**Figure 5E**). This suggests that the model, which is based on six variables, functions as an effective tool for predicting the progression from CIN III to CC.

An auxiliary validation of the model was performed using logistic regression, with data from 17 CIN III and 31 CC samples. ROC curves assessed the models, which had an AUC of 1 (**Figure 5F**). The equation for logistic regression is: p=EXP(X)/(1+EXP(X)), in this equation X=-35.611ARG2 + 27.690HSP90AA1 + 20.889ICAM1-22.393EZH2-0.11Macrophage M1 + 0.002Activated CD4 memory T cells+1.195, cutoff is 0.889, positive when P>cutoff, negative when p<cutoff. Correlation analysis in the experimental cohort showed that the correlation of the six predictors was less than 0.5, indicating that the six predictors have good application value (**Figure 5G**).

## 4 Discussion

CC is a prevalent gynecological tumor posing a significant threat to women’s health. The progression risk of CIN I to CC varies by grade: 60% of CIN I lesions resolve on their own, 11% advance to carcinoma *in situ*, and only 1% become invasive cancer. For CIN II and CIN III lesions, 5% and 12%, respectively, progress to invasive cancer (29). Early-stage CIN can be effectively treated with ablation (cryotherapy or thermal ablation) or excision (large ring excision or cold knife cone). However, it should not be ignored that simple cytological examination cannot accurately predict the potential of CIN to progress to CC. Thus, a predictive model for “inflammatory cancer transformation” is needed to aid clinicians in forecasting disease progression.

Persistent HR-HPV infection primarily causes CIN and CC, by using viral oncoproteins E6 and E7 to deactivate tumor suppressor genes p53 and pRB. This disruption of cell cycle control allows unchecked cervical cell division, promoting CIN and CC development and progression (30). Precisely because the body’s immune system is unable to completely clear the HPV virus, CC are infiltrated by a variety of TICs, which promote carcinogenesis (31, 32). Previous studies have shown that during the progression of CIN to CC, TICs in TIME are gradually dominated by CD8+ T cells and macrophages, and the CD4/CD8 ratio is reversed, which implies a decrease in the body’s anti-tumor immunity (33). On the other hands CC cells can induce the production of antigen-presenting cells, thus creating an immunosuppressive microenvironment that favors the survival of tumor cells (34). Meanwhile, TICRGs have an important role in tumorigenesis and tumor microenvironment formation, and their inactivation or upregulation may be associated with immune escape (35). It has been shown that TICRGs can progress CIN to CC by mediating inflammation and immune escape, and that certain methylated DNAs play a key role in controlling different transcriptional profiles in memory lymphocytes (36). In addition, these epigenetic mechanisms may involve antigen presentation, self/non-self-discrimination, and the balance between tolerance and autoimmunity (37). Therefore, identifying immune factors crucial to CIN’s progression to CC is vital for CC’s preventive diagnosis.

In this study, we identified six immune-related factors crucial for the progression from CIN to CC using public databases. The ARG2 gene, which encodes L-arginine acylase, is linked to cervical lesion progression and severity (38). Overexpression of ARG2 promotes CC cell proliferation and invasion while inhibiting apoptosis. This is accomplished by regulating L-arginine metabolism and modulating the tumor immune microenvironment. HSP90AA1, a molecular chaperone protein is significantly overexpressed in CC tissues compared to normal cervical tissues, especially in advanced CC (39), and is closely associated with the biological behaviors of tumor cells, including proliferation, metastasis, and drug resistance (40–42). The utilization of HSP90 inhibitors has been demonstrated to impede the proliferation and migration of CC cells, while simultaneously enhancing the sensitivity of radiation therapy (43). EZH2, a histone methyltransferase involved in chromatin modification and gene transcription, is associated with higher tumor malignancy, differentiation, and metastasis in CC (44–46),. The available evidence (47, 48) indicates that elevated EZH2 expression is associated with enhanced proliferation, invasion, and metastasis of CC cells, and is linked to a poor prognosis. ICAM1 is a cell surface protein that aids immune cells in identifying and destroying tumor cells. Its high expression boosts immune cell activity and infiltration, enhancing tumor surveillance. ICAM1 binds to ligands like LFA-1 and MAC-1, which play roles in inflammation and tumor metastasis (49–51).

M1 macrophages are immune cells that can participate in the regulation of the tumor microenvironment through the production of cytotoxins, chemokines, and inflammatory mediators, which play an important role in tumor growth and metastasis. It has been demonstrated (52) that in the tumor microenvironment, M1 macrophages may be polarized to M2 macrophages by certain factors secreted by tumor cells, including IL-10 and TGF-β, which consequently facilitate tumor growth and metastasis. Activated CD4 memory T cells state play a pivotal role in tumorigenesis, and these cells are capable of recognizing and attacking tumor cells, thereby playing a role in immune surveillance and tumor clearance (53). Accordingly, enhancing the efficacy of immune surveillance by activated CD4 memory T cells may represent a pivotal strategy for counteracting tumor immune evasion.

This study developed a prediction model using the random forest algorithm (54), evaluating its efficiency with the AUC under the ROC curve. The model achieved an AUC of 1 for internal training sets and 0.912 for testing sets. For validation cohort, the AUC was 0.864 for GSE7803 and 0.918 for TCGA/GTEx. For external validation (GSE39001, GSE149763, and GSE138080), the AUC was 0.703, 0.889 and 0.696. To validate the model’s effectiveness, this study tested six predictors in clinical cervical disease samples using mIHC experiments. These results served as external validation to confirm the model’s reliability. The findings indicate that the prediction model for “inflammation-cancer transition,” based on 4 TICRGs and 2 TIPs predictors, performed well in both internal and external cohorts.

Although the random forest algorithm has been widely applied in many fields, it still faces some challenges. Firstly, although random forests effectively reduce the risk of overfitting by integrating multiple trees, overfitting may still occur in situations with high data noise or small sample sizes. To solve this problem, model performance can be optimized by limiting the maximum depth of the tree, increasing the minimum number of sample splits or minimum number of sample leaves, and adjusting hyperparameters through cross validation. Secondly, the decision-making process of random forests is relatively complex, resulting in poor interpretability. To address this limitation, the contribution of variables can be evaluated through feature importance, or a visualization tool can be used to interpret a single decision tree to enhance the interpretability of the model. In addition, parameter selection and optimization of random forests is an important research direction, which can be automatically optimized through grid search or randomized search to improve model performance. Finally, random forests require a significant amount of computational resources when training multiple trees, especially when dealing with large-scale datasets or high-dimensional data, resulting in high computational costs. To solve this problem, a balance between performance and efficiency can be found by reducing the number of trees, or by dimensionality reduction and feature selection of the data to reduce the number of features, thereby reducing computational complexity. Through the above methods, the limitations of the random forest algorithm in practical applications can be effectively alleviated, further improving its performance and practicality.

In mIHC experiments, protein levels of HSP90AA1, ICAM1 and numbers of macrophage M1, and activated T cells CD4 memory increased with disease severity, consistent with bioinformatics findings. HSP90AA1 aids antigen release from cancer cells in TIME, while EZH2, STAT1, and ICAM1 hinder immune cell infiltration. Activated CD4 memory T cells promote inflammation and push CIN to CC. And M1 macrophages have anti-inflammatory effects. As the disease progresses, the anti-inflammatory effect of macrophages increases. At the same time, we can see that in the later stages of disease development, Macrophage M1 cannot play a good role in tissue repair, and Macrophage M1 will switch to Macrophage M2. The level of ARG2 protein increases as the disease progresses from CIN III to CC, supporting the view that ARG2 mRNA expression is significantly upregulated in women with cancer lesions. Conversely, EZH2 levels peaked at the CIN III stage and dropped significantly in CC, contradicting bioinformatics predictions of a steady increase, with the highest levels in CC. The cause of this discrepancy remains unknown. Meanwhile, we compared CIN III and CC groups and found significant differences in HSP90AA1, ICAM1, EZH2, ARG2, and macrophage M1, indicating these may be biomarkers for predicting CIN III’s progression to CC.

Although the model in this study has shown some predictive ability in preliminary validation, there are still limitations: 1. This study mainly relies on transcriptomic data, which may not fully capture the complexity of cervical cancer progression despite providing comprehensive information on gene expression levels. Therefore, future research should integrate multiple omics data to more comprehensively reveal the molecular mechanisms of cervical cancer. 2. The current research sample may have selection bias and lack patient data from different races, regions, and economic backgrounds. Therefore, future research should expand the sample size and include more diverse patient populations to ensure the universality and robustness of the model. 3. Current research is mainly based on correlation analysis and lacks support from functional experiments. Therefore, future research should further validate the functions of these genes through *in vitro* and *in vivo* experiments, and explore their specific mechanisms of action in the progression of CIN, in order to provide stronger theoretical basis for the early diagnosis and treatment of cervical cancer.

## 5 Conclusion

In this study, we developed a prediction model for the “inflammation-cancer transition” using six predictors: ARG2, HSP90AA1, EZH2, ICAM1, macrophage M1, and activated CD4 memory T cells. The model showed good predictive efficacy, as evaluated by the area under the ROC curve. The model showed strong predictive performance in both validation and experimental cohorts, suggesting it can somewhat predict CIN progression to CC. Expression levels of HSP90AA1, EZH2, ICAM1, and macrophage M1 increased progressively across the four cervical lesion stages, with significant intergroup differences. These findings indicate that the biomarkers could be useful in clinical settings. ARG2 showed a steady decline across cervical lesion stages, suggesting it might protect against CC. This model could help predict patient immune status and disease progression, enabling timely interventions at the CIN or early CC stages. Additionally, it may provide clinicians with new insights for diagnosing cervical diseases.

## Data Availability

The original contributions presented in the study are included in the article/**Supplementary Material**. Further inquiries can be directed to the corresponding authors.
