# Supplementary material for: A predictive model for the transformation from cervical inflammation to cancer based on tumor immune-related factors
Source: Front Immunol. 2025 Apr 25;16:1532048. doi: 10.3389/fimmu.2025.1532048 (PMC12062085; doi:10.3389/fimmu.2025.1532048)
Supplement: Supplementary file 1 [file Table1.docx]

Supplemental Table 1 Sources of data and number of sample cases at each stage of cervical pathology

| Data Sets | Purpose of data | Number of sample cases (cases) | | |
| --- | --- | --- | --- | --- |
|  |  | Normal | CIN | CC |
| GES63514 | Training cohort | 24 | 62 | 28 |
| GSE7803 | Validation cohort | 10 | 7 | 21 |
| TCGA、GTEx | Validation cohort | 13 | 0 | 306 |
| GSE39001、GSE149763、GSE138080 | External verification queue | 22 | 18 | 56 |

Supplemental Table 2 Organizational sample size and two model experimental validation sample sizes

| Experimentally validated models | Number of tissue sample cases (cases) | | | | Total number (cases) |
| --- | --- | --- | --- | --- | --- |
|  | Normal | CIN II | CIN III | CC |  |
| Number of cases | 21 | 5 | 17 | 31 | 74 |

| Supplemental Table 3 31 genes related to cancer immune cycle with non-zero coefficients and their correlation coefficients | |
| --- | --- |
| TICRGs | Correlation coefficient |
| HSPA2 | -0.03199 |
| HSP90AA1 | 0.03916 |
| HSP90AB1 | 0.00470 |
| IFNA1 | -0.84716 |
| IL1A | 0.02355 |
| IL1B | 0.00784 |
| TLR3 | -0.0098 |
| TLR5 | -0.035921 |
| TLR6 | 0.16930 |
| TLR9 | -0.17962 |
| CD247 | -0.00030 |
| TNFSF9 | -0.0110 |
| TNFSF4 | 0.08542 |
| CD27 | -0.0596 |
| TNFSF14 | -0.14031 |
| SLAMF6 | -0.00782 |
| SLAMF9 | 0.12678 |
| CD160 | -0.00855 |
| IL12A | 0.02442 |
| IL12B | -0.07973 |
| CCL19 | 0.01364 |
| CXCL16 | 0.00039 |
| CCL5 | -0.01338 |
| CXCR2 | -0.03681 |
| CXCL5 | -0.03878 |
| CXCL12 | 0.01195 |
| CCL1 | -0.21013 |
| STAT1 | 0.01688 |
| ICAM1 | 0.02515 |
| EZH2 | 0.0229 |
| ARG2 | -0.02191 |
