# Supplementary figures and images for: A predictive model for the transformation from cervical inflammation to cancer based on tumor immune-related factors
Source: Front Immunol. 2025 Apr 25;16:1532048. doi: 10.3389/fimmu.2025.1532048 (PMC12062085; doi:10.3389/fimmu.2025.1532048)

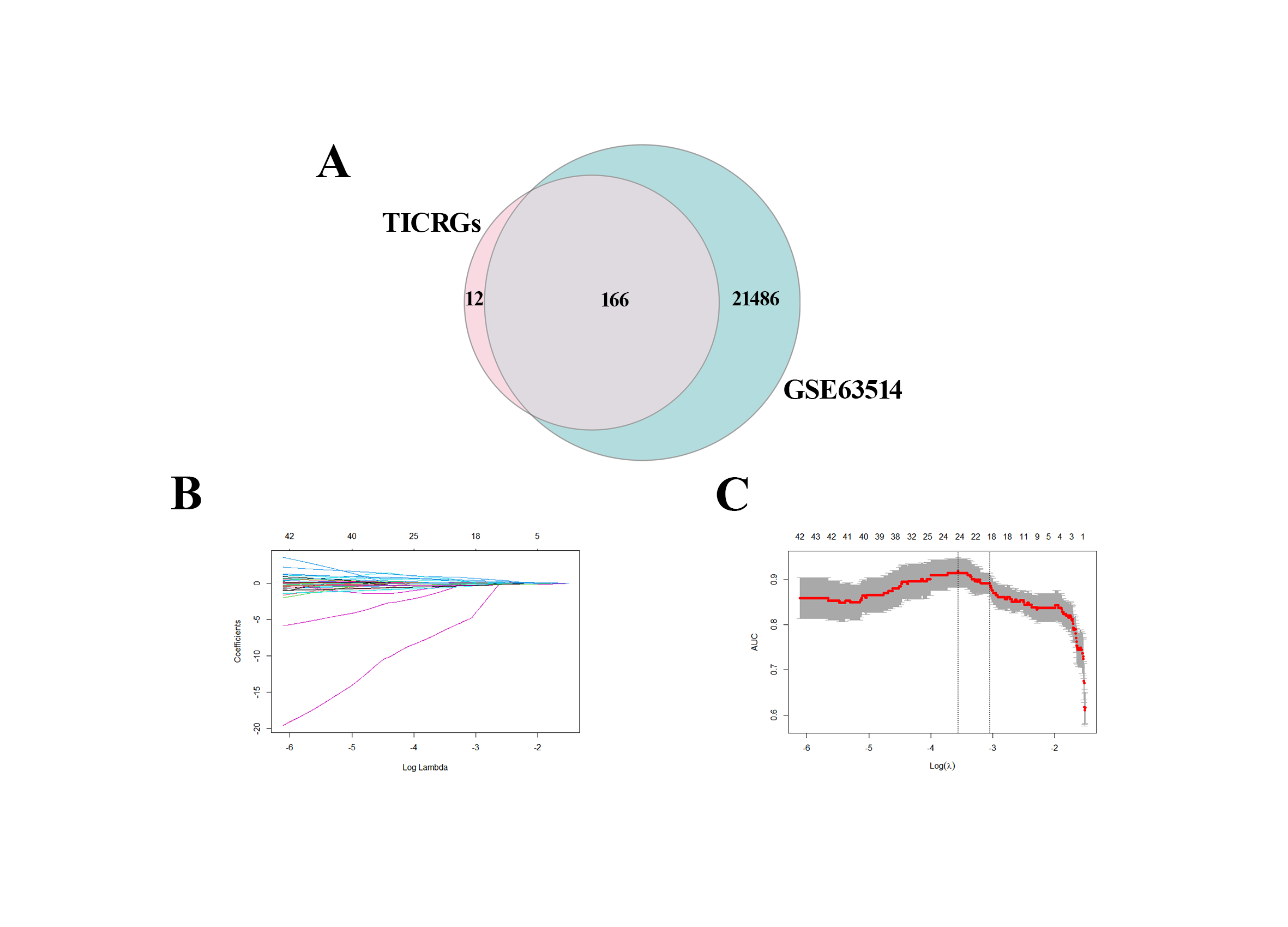

Supplement: Supplementary file 2 [file Image1.tif]
